# Molecular docking analysis of COX-2 for potential inhibitors

**DOI:** 10.6026/97320630016753

**Published:** 2020-10-31

**Authors:** Jayaraman Selvaraj, Veeraraghavan Vishnupriya, Hussain Sardar, Janardhana Papayya Balakrishna, Josephine Rex, Surapaneni Krishna Mohan, Periyasamy Vijayalakshmi, Rajagopal Ponnulakshmi

**Affiliations:** 1 Department of Biochemistry, Saveetha Dental College and Hospitals, Saveetha Institute of Medical and Technical Sciences, Saveetha University, Chennai - 600 077, India; 2 Department of Biotechnology, Government Science College, Chitradurga-577501, Karnataka, India; 3 Department of Stem Cell Biology, Stellixir Biotech Pvt Ltd, No.V-31, 2nd floor, 10th Main Road, Peenya 2nd Stage Industrial Area, Bangalore - 560058, Karnataka, India; 4 Department of Biochemistry, Panimalar Medical College Hospital & Research Institute, Varadharajapuram, Poonamallee, Chennai - 600 123, India; 5 Department of Biochemistry and Department of Clinical Skills & Simulation, Panimalar Medical College Hospital & Research Institute, Varadharajapuram, Poonamallee, Chennai - 600 123, India; 6 PG & Research Department of Biotechnology & Bioinformatics, Holy Cross College (Autonomous), Trichy- 620002, Tamil Nadu, India; 7 Central Research Laboratory, Meenakshi Academy of Higher Education and Research (Deemed to be University), Chennai-600 078, India

**Keywords:** Breast cancer, COX-2, alkaloids, molecular docking

## Abstract

Cyclooxygenase-2 (COX-2) is liked with breast cancer. Therefore, it is of interest to design and develop new yet effective compounds against COX-2 from medicinal plants such as the natural alkaloid compounds. We document the optimal binding features of aristolochicacid with COX-2 protein for further consideration.

## Background

Breast cancer is the most common malignancy among women in the developed countries and the main cause of female cancer-related deaths. In the past 15 years, the prevalence of breast cancer has risen by two thirds. In recent years, the worldwide incidence of breast cancer has increased rapidly. The situation in Eastern India also shows the same pattern [1]. Unfortunately, breast cancer patients are at a 3-to 5-fold greater risk of second breast tumor compared to the population [2]. Nearly 20 % of patients have a markedly decreased chance of long-term survival [3].

The prognosis of breast cancer depends on various biological and molecular factors [4]. Cyclooxygenase ( COX) type of enzyme is essential for the conversion of arachidonic acid into prostaglandins. Cyclooxygenase-1 (COX-1) is mainly expressed at a consistent rate through out cell cycle in certain tissues. Inducible isoform Cyclooxygenase-2 (COX-2) can be over-expressed in breast cancer [5]. Several research articles indicate that COX-2-derived metabolites that contribute towards the development of tumor survival, pre - cancerous hyperproliferation, tumor development, transformation, invasion, and metastatic spread [6] and COX-2 has been shown to be over expressed in many human malignant tumours [7]. Prostaglandins induce the occurrence and activation of aromatase [8], an enzyme that inhibits androgen to estrogen. Estrogen can enhance the growth of cancerous cells by activating the estrogen receptor (ER) and target genes. This is one mechanism by which COX-2 may promote breast cell proliferation and angiogenesis [9]. COX-2 activation stimulation in ERnegative breast cells can occur through protein kinase C (PKC) and/or RAS / MAPK pathways. COX-2 expression is consistent with angiogenesis and lymph node metastases in human breast cancer [10].

Alkaloids are a very complex group of compounds that comprise a ring structure and a nitrogen atom. In most situations, the nitrogen atom is found inside the heterocyclic ring structure [11]. As well, some alkaloids demonstrate important biological activity, such as the easing action of ephedrine for asthma, the analgesic action of morphine, and the anticancer effects of vinblastine [12]. In addition, alkaloids are one of the most essential active ingredients in herbal plants, but some of these compounds have been already actively implemented into chemotherapy drugs, such as camptothecin (CPT), the popular Topoisomerase I (TopI) inhibitor [13], and vinblastine, which associates with tubulin. Therefore, it is of interest to design and develop effective compounds against COX-2 from medicinal plants such as the natural alkaloid compounds (Table 1 -see PDF).

## Materials & Methods:

### Obtaining the crystal structure of the target COX2:

To prepare the COX-2 structure, the crystal structure was taken from the Protein Data Bank (PDB_ID: 5KIR). Heteroatoms were eliminated from the active site and the A chain was chosen for docking studies. Hydrogen atoms have been added to the enzyme. Docking experiments were performed using the COX-2 binding site.

### Ligand preparation:

In our present research, chosen ten natural alkaloid compounds were obtained from the pubchem database in SDF format and translated into a PDB file format using the Online Smile Translator. Energy minimization of ligands was completed using ChemBio 3D Ultra 12.0, based on the method stated. Energy reduced ligand structures have been transferred to HEX applications.

### Active site identification:

The binding site identification was carried out using CSATp server. This server recognizes the atoms that line pockets, pocket openings, and hidden cavities; the amount and region of pockets and cavities; and the position and diameter of the mouth openings.

### Molecular Docking Analysis:

Hex8.0 program (http:/hex.loria.fr) has been used for docking experiments to identify potential interactions between the COX-2 protein and selected alkaloid compounds. The Interactive Molecular Graphics System is designed to measure and view potential protein and ligand docking modes with the best scores and to discover the drug-receptor complex with the lowest free energy [14].

## Results and Discussion:

**Figure 1:**
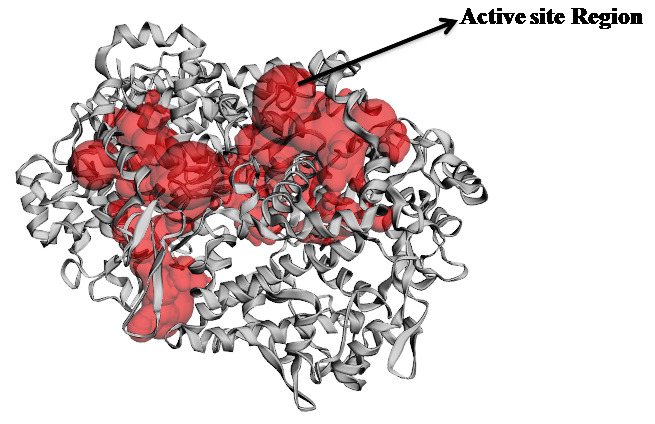
Predicted active site Region using CASTp. Red colour sphere indicates the predicted active site region.

The binding site residues in the COX-2 structure were retrieved from the CAST p system. The findings indicate that ASP-125, PRO-127, TYR-130, ASP-133, TYR-134, GLY-135, VAL-155, PRO-156 and ASP-157 (Figure 1) serve as binding residues in the COX-2 protein. The main objective of the molecular docking analysis of alkaloid compounds is to find a molecule that displays a strong binding affinity to the target protein COX-2 and also to build a stable complex. Thus, the frequency of the interaction between the two molecules can be determined by the use of score functions. The higher the ranking, the more stable the complex is. As a result, molecular docking can be viewed as an optimization issue that defines which ligand has the 'best fit' orientation towards the target protein. The molecular docking study was performed between the target molecule COX-2 and the alkaloid compounds using Hex 8.0.0 software. Hex software helps the receptor molecule to rotate on the Z axis. From the results obtained, Aristolochicacid has a higher energy content with COX-2 compared to any other molecule.

**Figure 2:**
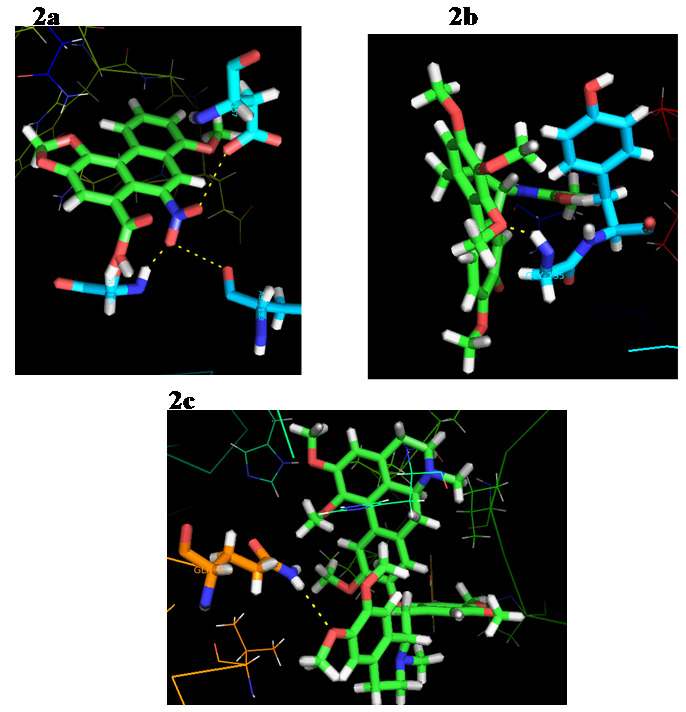
Molecular interaction of best three compounds. Green colour sticks representation is a compound and yellow colour dotted line represents h-bond interaction. Interactions of the protein target COX-2 with (a) aristolochicacid (b) colchicine and (c) oxyacanthine.

The docking ratings on the comparison represent the best docking candidates for COX-2 (Table 2 - see PDF). The docking results were produced and shown using pymol (Figure 2). As shown by the test, the docking score for the selected compounds is similar enough and thus reflects their maximal activity against COX-2. Depending on the score parameter, we picked the best three compounds from a total of 10 compounds. Based on the docking ranking, the lowest e-value observed at the docking of Oxyacanthine with COX-2 was-353.95 KJ / mol. The maximum E values found in COX-2 and Aristolochicacid were-275.54 KJ / mol. Active chemical interaction takes place between the COX-2 protein and the selected alkaloid molecule by Van der Waals forces, electrostatic and hydrogen bonding. The findings showed that all the investigative molecules had higher energy values on the COX-2 receptor, which means that these alkaloids have greater affinity and steric compatibility with COX-2. Aristolochicacid molecule displays a strong pattern of COX-2 binding in terms of energy-275.54. It can serve as a possible drug candidate against breast cancer relative to all other compounds.

## Conclusion:

We document the optimal binding features of aristolochicacid with COX-2 protein for further consideration in the context of breast cancer.
